# Diagnostic performance of an automated microscopy and pH test for diagnosis of vaginitis

**DOI:** 10.1038/s41746-023-00815-w

**Published:** 2023-04-13

**Authors:** Ahinoam Lev-Sagie, Doris Strauss, Avraham Ben Chetrit

**Affiliations:** 1 Faculty of Medicine, Hebrew University of Jerusalem, Jerusalem, Israel; 2 Clalit Health Organization, Jerusalem, Israel

**Keywords:** Diagnosis, Urogenital reproductive disorders, Medical imaging

## Abstract

Vaginitis is a common gynecological problem, nevertheless, its clinical evaluation is often insufficient. This study evaluated the performance of an automated microscope for the diagnosis of vaginitis, by comparison of the investigated test results to a composite reference standard (CRS) of wet mount microscopy performed by a specialist in vulvovaginal disorders, and related laboratory tests. During this single-site cross-sectional prospective study, 226 women reporting vaginitis symptoms were recruited, of which 192 samples were found interpretable and were assessed by the automated microscopy system. Results showed sensitivity between 84.1% (95%CI: 73.67–90.86%) for *Candida albicans* and 90.9% (95%CI: 76.43–96.86%) for bacterial vaginosis and specificity between 65.9% (95%CI: 57.11–73.64%) for *Candida albicans* and 99.4% (95%CI: 96.89–99.90%) for cytolytic vaginosis. These findings demonstrate the marked potential of machine learning-based automated microscopy and an automated pH test of vaginal swabs as a basis for a computer-aided suggested diagnosis, for improving the first-line evaluation of five different types of infectious and non-infectious vaginal disorders (vaginal atrophy, bacterial vaginosis, *Candida albicans* vaginitis, cytolytic vaginosis, and aerobic vaginitis/desquamative inflammatory vaginitis). Using such a tool will hopefully lead to better treatment, decrease healthcare costs, and improve patients’ quality of life.

## Introduction

Vulvovaginal symptoms, collectively termed vulvovaginitis, are one of the most common reasons for gynecological consultation^1^. Most women will experience at least one episode of vaginal infection during their lifetime, characterized by discharge, itch, burning, or odor^2^. Vulvovaginitis is caused by various infectious and non-infectious conditions that may present as an acute, short-term complaint, or as a chronic disorder. Vaginitis conditions, and more so in their recurrent or chronic forms, have a negative impact on women’s health-related quality of life^3^ contributing to a major negative impact on self-esteem and sexual relationships^4^. Mixed vaginitis, where at least two types of vaginitis are present, is reported in 4.4% to 35% of evaluated patients^5^.

Diagnosis of the specific cause of vaginitis based on clinical presentation is limited, due to the similarity of symptoms and signs, such as itching, erythema, malodor, and vaginal discharge. Therefore, to obtain an accurate diagnosis additional procedures or diagnostic tests are required. Clinical guidelines suggest that pH and wet mount microscopy of fresh vaginal samples constitute the in-clinic standard practice for the diagnosis of vaginitis^2,6–8^. However, the actual level of use of these two measures is low. Hillier et al. reported that among women with vulvovaginitis symptoms, microscopy was conducted in only 17.4% of 281 visits^9^. An earlier study showed that microscopic assessment of vaginal discharge was not performed in 37% of 150 clinic visits^10^, and 42% of 50 different physicians did not perform microscopy as part of the evaluation of vaginitis^10^. Additionally, in >90% of office visits, pH measurement of vaginal discharge was not performed^10^. A review of 149,934 American patients’ files found that over 60% did not include procedure codes for any form of vaginitis diagnostic testing^11^. High rates of antifungal and antibacterial use were found, suggesting common empiric treatment, and likely over-prescription of antifungal and antibacterial medications^11^. A study from the Netherlands found that only 16% (61/380) of GPs reported “always” or “often” using microscopy to diagnose vulvovaginal candidiasis, while only 7.9% (30/380) reported “always” or “often” using culture for the same purpose^12^. Other reports found high levels of misdiagnosis of bacterial vaginosis (BV) and vulvovaginal candidiasis (VVC), regardless of utilization of microscopy, suggesting inadequate use of microscopy as a possible explanation^13^. Underutilization of these simple in-clinic tests often leads to inadequate treatment^9,10,14^, with up to 47% of patients receiving one or more inappropriate prescriptions^9^, as well as treatment without adequate evaluation in 54% of visits, implying appropriate treatment in fewer than half of the cases^10^.

An insufficient in-clinic evaluation may result from the absence of suitable equipment (i.e., microscope), lack of proper training in the preparation and interpretation of a wet mount, time constraints, and lack of awareness that these measures allow improved detection of the causes of vaginitis^1^. As an alternative to microscope usage, providers can send vaginal samples for laboratory evaluation. Nevertheless, laboratory tests are aimed at detecting only infectious etiologies and cannot detect noninfectious conditions such as vaginal atrophy, desquamative inflammatory vaginitis (DIV, also termed aerobic vaginitis, AV), and cytolytic vaginosis. In addition, these methods are not available in many clinical settings, are expensive, time-consuming, and do not provide timely results, as many of these tests require a process that lasts from several hours^15^ to several days, thus not allowing a point-of-care diagnosis.

Laboratory methods include cultures, a multiplex polymerase chain reaction (PCR) panel, and nucleic acid amplification testing (NAAT)^6^. Some rapid point-of-care tests are available^16,17^, nevertheless, these tests are only for a subset of the related conditions, including BV and trichomoniasis. Each test can only diagnose a single cause, necessitating the conduction of multiple tests, thus increasing the turnover time and cost. Collectively, these findings suggest that current clinical practice is sub-optimal and indicate the need for a diagnostic modality that is efficient, cost-effective, allows for a wide range of diagnoses, and is usable during the clinic visit^18,19^.

The investigational test evaluated in this study is the GYNI™ rapid point-of-care system (GynTools, Israel), an automated in-vitro diagnostic system, intended to aid in the diagnosis of vaginitis in symptomatic women, by qualitatively detecting the following vaginitis conditions or pathogens at the point-of-care: (1) BV, (2) *Candida albicans* vaginitis (CA), (3) *Candida* non-albicans vaginitis (NAC), (4) *T. vaginalis*, (5) vaginal atrophy (also referred to as atrophic vaginitis or genitourinary syndrome of menopause, GSM), (6) aerobic vaginitis/desquamative inflammatory vaginitis (AV/DIV), and (7) cytolytic vaginosis (CV).

The objective of this study was to evaluate the diagnostic performance of the investigational test in qualitatively detecting different vaginal disorders, by comparison to a composite reference standard (CRS) of wet mount microscopy performed by a specialist in vulvovaginal disorders (the first author, ALS) and laboratory tests of CHROMagar™ candida culture and sexually transmitted infection (STI) multiplex PCR for detection of trichomoniasis.

## Results

### Performance of the investigational test

A total of 226 women with vaginitis symptoms were recruited between December 2020 and October 2022. The majority were Caucasians, representing the Israeli population.

**Fig. 1:**
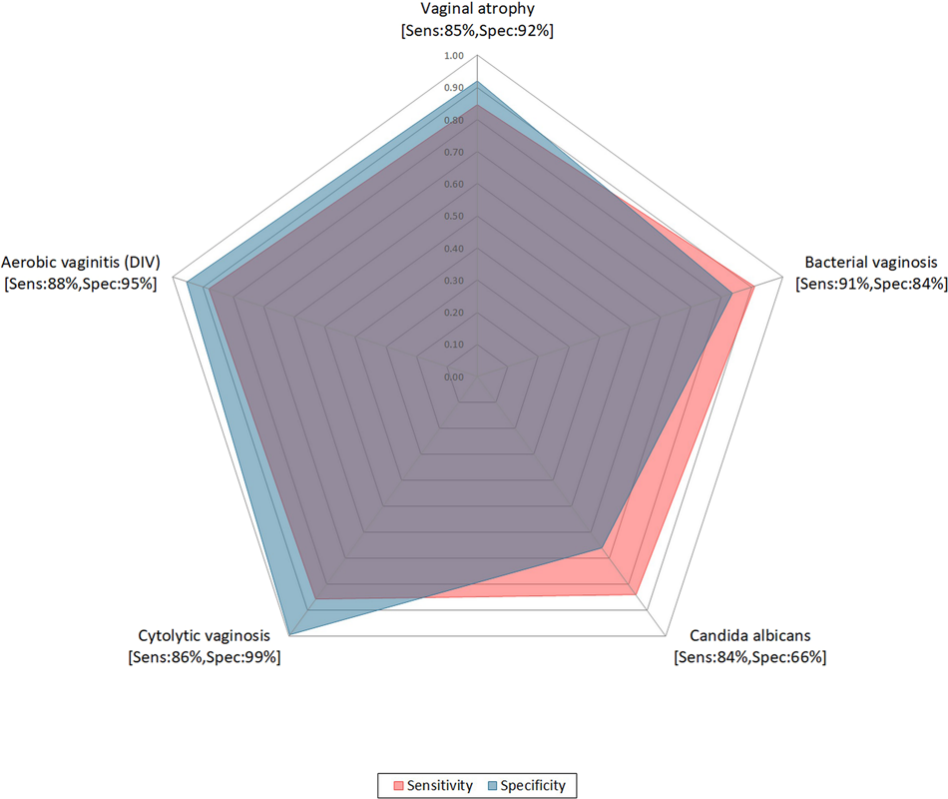
The investigational test diagnostic performance chart. *T. vaginalis* and *Candida* non-*albicans* are excluded due to a low number of cases.

Thirty-four cases were excluded in accordance with the exclusion criteria of an uninterpretable sample. The performance of the investigational test for the detection of each condition based on a total of 192 included cases is shown in Table 1 and Fig. 1.

**Table 1:** Diagnostic Performance of GYNI™ test compared to a CRS of culture, PCR, and wet mount microscopy.

Condition		Overall Accuracy	Sensitivity	Specificity	PPV^b^	NPV^c^
Vaginal Atrophy	% (n/N)	91.15% (175/192)	84.62% (22/26)	92.17% (153/166)	62.86% (22/35)	97.45% (153/157)
	95% CI^a^	[86.28%; 94.40%]	[66.47%; 93.85%]	[87.06%; 95.37%]	[46.34%; 76.83%]	[93.63%; 99.00%]
Bacterial Vaginosis	% (n/N)	84.90% (163/192)	90.91% (30/33)	83.65% (133/159)	53.57% (30/56)	97.79% (133/136)
	95% CI	[79.15%; 89.27%]	[76.43%; 96.86%]	[77.12%; 88.59%]	[40.70%; 65.98%]	[93.72%; 99.25%]
*Candida albicans*	% (n/N)	72.40% (139/192)	84.06% (58/69)	65.85% (81/123)	58.00% (58/100)	88.04% (81/92)
	95% CI	[65.68%; 78.23%]	[73.67%; 90.86%]	[57.11%; 73.64%]	[48.21%; 67.20%]	[79.85%; 93.19%]
Cytolytic Vaginosis	% (n/N)	98.44% (189/192)	85.71% (12/14)	99.44% (177/178)	92.31% (12/13)	98.88% (177/179)
	95% CI	[95.51%; 99.47%]	[60.06%; 95.99%]	[96.89%; 99.90%]	[66.69%; 98.63%]	[96.02%; 99.69%]
AV/DIV	% (n/N)	94.27% (181/192)	88.00% (22/25)	95.21% (159/167)	73.33% (22/30)	98.15% (159/162)
	95% CI	[90.03%; 96.77%]	[70.04%; 95.83%]	[90.83%; 97.55%]	[55.55%; 85.82%]	[94.70%; 99.37%]

^a^*CI* confidence interval.

^b^*PPV* positive predictive value.

^c^*NPV* negative predictive value.

### Performance of the specialist

The results for the specialist diagnosis, compared to the CHROMagar^™^ *Candida albicans* culture tests were of an overall accuracy level of 93.1% (189/203) and a sensitivity and specificity of 86.7% (72/83) and 97.5% (117/120), respectively, and κ of 0.855 (0.782–0.927), as shown in Table 2.

**Table 2:** Diagnostic performance of physician diagnosis compared to lab culture for Candidiasis.

Condition		Overall Accuracy	Sensitivity	Specificity	PPV^b^	NPV^c^
*Candida albicans*	% (n/N)	93.10% (189/203)	86.75% (72/83)	97.50% (117/120)	96.00% (72/75)	91.41% (117/128)
	95% CI^a^	[88.76%; 95.85%]	[77.81%; 92.44%]	[92.91%;99.15%]	[88.89%; 98.63%]	[85.27%; 95.13%]

^a^*CI* confidence interval.

^b^*PPV* positive predictive value.

^c^*NPV* negative predictive value.

### Condition distribution

The number of cases found of each condition by the gold standard method of either the expert or the laboratory results are as follows (including coinfections): *Candida albicans* vaginitis: 69, BV: 33, vaginal atrophy: 26, AV/DIV: 25, cytolytic vaginosis: 14, other conditions, including *T. vaginalis* and *Candida* non *albicans:*28. In 17 cases no definite diagnosis was made by the CRS.

**Fig. 2:**
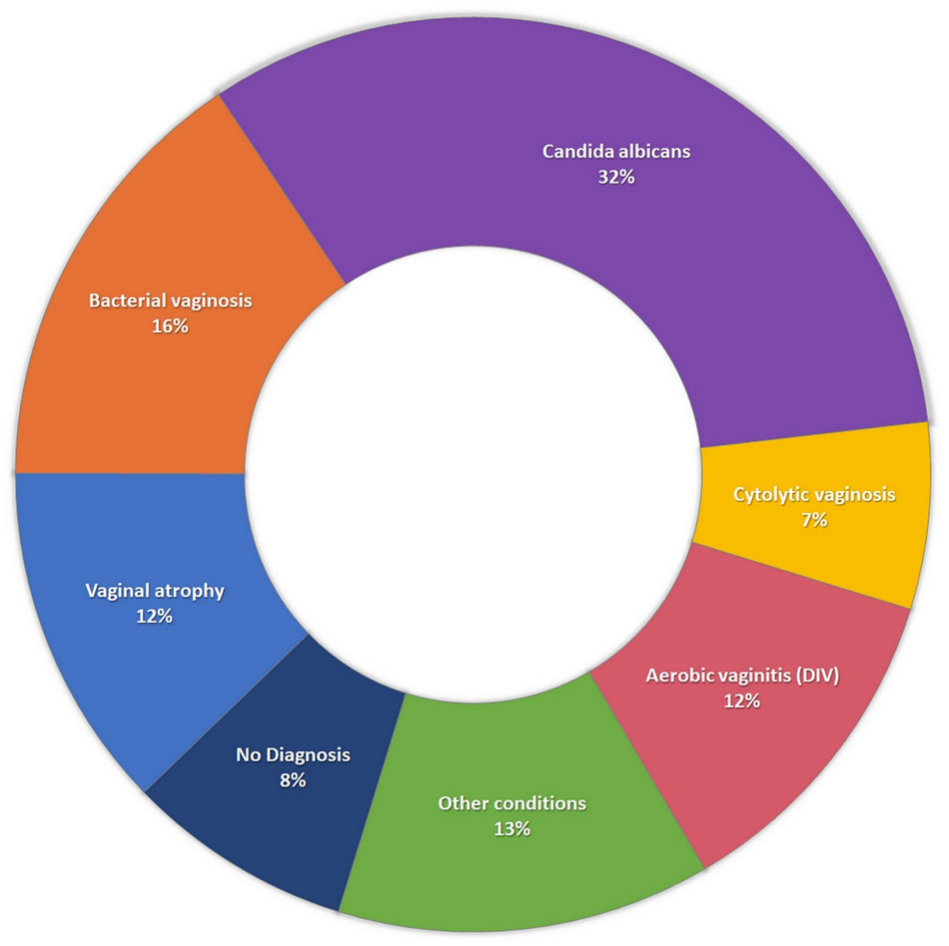
The percent of cases by condition. Every diagnosed condition was separately counted. Reflecting 195 diagnosed conditions/192 patients including 22 cases of coinfection and 17 cases of no established diagnosis.

The percent of cases by condition is presented in Fig. 2.

### Coinfections

We found 22 cases (11.5%) where the CRS indicated a coinfection, i.e., presence of more than one condition^20^. 12 cases were for a combination of BV and *Candida albicans* vaginitis. The other combinations were *Candida albicans* and *Candida* non-*albicans* (3), BV and DIV (2), *Candida albicans* and DIV (2), cytolytic vaginosis and *Candida* non-*albicans* (1), atrophy, *Candida albicans* and *Candida* non-*albicans* (1), BV and *T. vaginalis* (1).

### Symptoms distribution

**Fig. 3:**
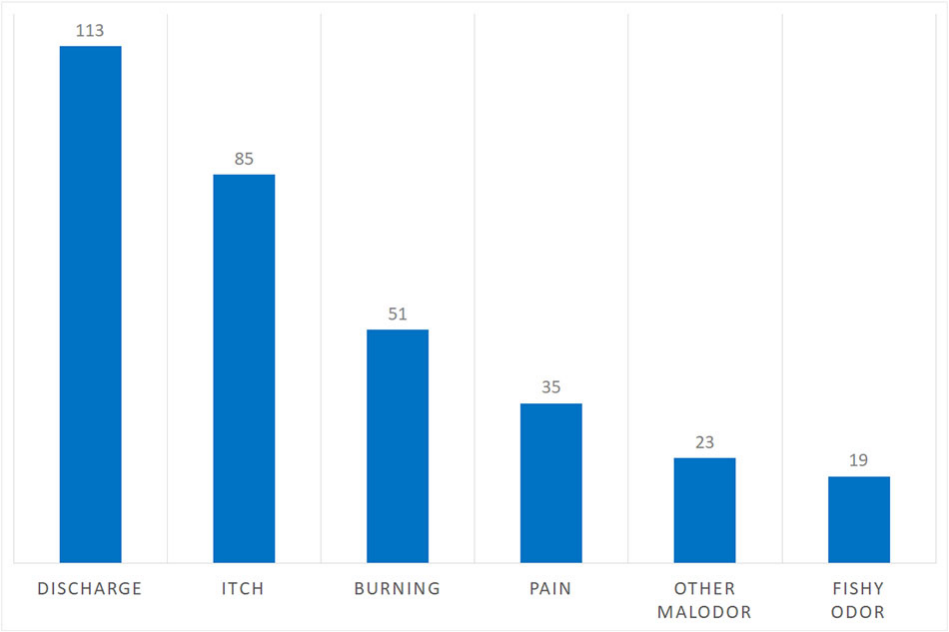
The distribution of patient-reported vaginal symptoms. The bars depict the number of patients reporting each symptom.

The distribution of patient-reported vaginal symptoms is presented in Fig. 3.

## Discussion

This study aimed to evaluate the performance of the investigational test in diagnosing seven vaginitis conditions, from a single swab, in a clinical setting.

The results for two of the seven conditions (*Candida* non *albicans* and *T. vaginalis*) were not reported due to an insufficient number of cases. For the remaining five conditions, sensitivity and specificity ranged from 84.1%/65.9%, respectively for *Candida albicans* and up to 90.9%/83.6% for BV. These results are superior to those reported for in-clinic testing (Amsel’s test, potassium hydroxide preparation, and wet mount) performed during routine clinic visits^21^.

It should be noted that samples of more than 10% of recruited patients were excluded: samples obtained from the first 30 patients were uninterpretable due to a camera stabilization problem that was identified and resolved by a scanner software update. One patient used a topical external ointment, a temporary server error occurred in one case, and reading was not available in one case due to a scanner horizontal movement malfunction.

Studies reported that at least 30% of women presenting with vaginal complaints do not receive a diagnosis after a comprehensive workup^1^. Furthermore, the presence of coinfections makes accurate diagnosis of vaginitis even more challenging. It was previously shown that clinician diagnosis of BV based on microscopy was less accurate when *T. vaginalis* and/or *Candida spp*. were also detected in the sample by the reference methods^22^. In addition, studies comparing diagnostic accuracy of providers to that of laboratory results, showed a high proportion of disagreement between the practitioners’ diagnoses and the laboratory diagnoses^10,14,23^. Nyirjesy et al conducted a survey among 333 physicians in order to measure awareness of vaginitis clinical guidelines and the use of in-office diagnostic tools^24^. The study found limited awareness of recommended diagnostic practice guidelines, and limited access to all three point-of-care tools (microscope, pH test strips and KOH solution)^24^.

Detecting the etiology of both infectious and non-infectious vaginal disorders provides an unprecedented diagnostic spectrum. To our knowledge, our results are the first ever reported for any automated tool to aid in diagnosing either vaginal atrophy or cytolytic vaginosis. In these cases, the diagnosis is based on cytologic characteristics and pH level. Correct diagnosis of vaginal atrophy is of high significance, since according to the 2020 position statement of the North American Menopause Society, GSM affects approximately 27% to 84% of postmenopausal women and can significantly impair health, sexual function, and quality of life, but only a minority of them seek help or are offered assistance by their physicians^25^.

Unlike laboratory methods such as Gram stain and culture which may be highly subjective to sampling, transport conditions, and technical proficiency, and may have prolonged turnaround times^26^, the evaluated investigational test provides fast in-clinic results from a single specimen within 5 minutes from scan initiation. This is done by automating the entire recommended practice of reported symptoms consideration, pH measurement, slide preparation, and slide scanning, and by harnessing deep-learning computer-vision image classification to support the provider’s diagnosis.

In various primary care settings, multiple reasons preclude an accurate diagnosis of vaginitis during the clinic visit, including the lack of an experienced microscopist. In addition, in some countries the first-line physician diagnosis for vulvovaginitis is made by a gynecologist and in others (for example, the United Kingdom and the Netherlands), it is a GP. The presented results suggest that using an in-office automated tool that relies on a combination of inputs (automated microscopy, automated pH, and patient’s reported symptoms) can improve patient’s evaluation and treatment, regardless of caregivers training and skills. This may result in reducing empirical treatment, as well as resolving both subjective intra and inter-observer variability between caregivers who do perform microscopy.

One major limitation of this study is the relatively small sample size, especially for the less prevalent disorders. Another limitation is that the deep-learning software model that provides the computer-vision classification was trained on images that were classified and tagged by a single physician, whose diagnosis also acted as one of the reference methods in this study. Better evaluation of true performance for BV is constrained by the known limitations of the Amsel criteria which demonstrated only 81.0% positive percent agreement and 86.0% negative percent agreement compared with positive (7–10) and negative (0–3) Nugent score^27^.

Given the above-described limitations of the current point-of-care diagnosis of vaginitis, the investigational test presents a potential for a marked improvement in the first-line evaluation of vaginitis and will hopefully guide a more appropriate treatment while decreasing healthcare costs and improving patients’ quality of life.

## Methods

### Outline

This prospective cross-sectional study evaluated women reporting vaginal symptoms, seen at a single designated clinic for vulvovaginal disorders at the Clalit Health Organization, Jerusalem, Israel. All patients were examined, diagnosed, and treated by the same provider (the first author, ALS). Inclusion criteria were (i) women with vaginal complaints: discharge, malodor, itch, burning, pain, or dryness, (ii) 18 years old and above. Exclusion criteria included (i) patients unfit to provide informed consent, (ii) an uninterpretable sample (e.g., patients who used vaginal creams or lubricants before the visit, recent or current bleeding, or insufficient sampling material).

During the gynecological exam, vaginal discharge samples were taken for pH levels, wet-mount microscopy which was conducted immediately in the clinic using Olympus CX31 microscope, vaginal cultures (for bacteria and CHROMagar^™^ candida), and trichomonas PCR, per standard of care in the clinic. An additional sample was taken for the investigational test diagnosis using a swab with a soft cytobrush head (Fig. 1), pulled through a dedicated cartridge, and scanned in the GYNI™ investigational test table-top scanner in the clinic.

Patients were diagnosed by the physician using wet-mount microscopy and were treated according to her recommendation. The investigational test diagnosis was stored in the cloud and blinded to the physician to prevent bias. Each patient was identified using a numerical code and an investigational test number was generated by the application. Laboratory results were recorded only by the patient’s code number, without any identifying details such as name or personal identification number. The comparators used as the gold standard for each of the assessed conditions studied are shown in Table 3. Results of the three methods, the physician’s wet-mount diagnosis, the laboratory findings, and the investigational test, were summarized and compared by the chief investigator (the last author, ABC).

**Table 3:** Composite reference standard (CRS) comparators.

Condition	Lab Comparator	Specialist Comparator
Bacterial vaginosis	–	Amsel criteria (including typical microscopy as an imperative criterion, i.e., presence of clue cells)
*Candida albicans*	CHROMagar^TM^ culture test	WMM^a^
*Candida* non-*albicans*	CHROMagar^TM^ culture test	WMM^a^
Trichomonas	Multiplex PCR^b^ (SeeGene Allplex STI-EA)	WMM^a^
Vaginal Atrophy (GSM^c^)	–	WMM^a^
Aerobic vaginitis (DIV)	–	WMM^a^ showing parabasal cells and >1:1 inflammatory to squamous cells; purulent discharge not explained by other entities; and elevated pH^34^
Cytolytic vaginosis	–	WMM^a^ showing increased lactobacilli, cytolysis with bare or naked intermediate nuclei. pH < 3.8 and a negative yeast culture^35^

^a^Wet Mount Microscopy

^b^Polymerase Chain Reaction

^c^Genitourinary syndrome of menopause

Clinical diagnostic criteria applied by the CRS: BV was diagnosed based on 3 out of 4 Amsel’s criteria including typical microscopy as an imperative criterion, i.e., presence of clue cells and coccobacillary microbiota; *C. albicans* vaginitis was diagnosed based on identification of *C. albicans* in culture with or without identification of hyphae/spores on microscopy; *Candida* non-*albicans* vaginitis was diagnosed based on its identification in culture with or without identification of hyphae/spores on microscopy; *T. vaginalis* was diagnosed based on its identification by Multiplex PCR or by identification of a motile parasite on WMM; Vaginal atrophy was diagnosed based on typical appearance of vaginal mucosal thinning, erythema, dryness, pH measurement>5 and WMM showing abundant parabasal cells; Aerobic vaginitis (DIV) was diagnosed based on combination of symptoms (at least one of the following: vaginal discharge, dyspareunia, pruritus, burning, irritation), vaginal inflammation (spotted rash, erythema, erosion), vaginal pH >4.5 and WMM showing increased numbers of parabasal and inflammatory cells (leukocyte to epithelial cell ratio greater than 1:1), PCR should be negative to *T. vaginalis*, *N. gonorrhea* and *C. trachomatis*, and culture rules out group A streptococcus and candida; Cytolytic vaginosis was diagnosed based on WMM showing increased lactobacilli, broken epithelial cells (cytolysis) with bare or naked nuclei, pH<3.8 and a negative yeast culture.

The sample size for this study was calculated for estimating the overall accuracy (the percent agreement on the diagonal between the investigational test and the reference diagnosis) via the level of precision required for the estimate. The level of precision is measured by the half-width of the 95% confidence interval around the proportion of interest. We calculated based on Hajian-Tilaki K^28^ that an accuracy level of at least 90% with a confidence interval half-width of 5% can be estimated with a minimum sample size of 139 patients. Recruitment continued to a larger sample size to obtain reasonable representation of each of the included conditions.

The results of the investigational test were compared individually to the CRS comparator of the specialist wet mount results, the candida cultures, and the STI PCR panel for detection of *T. vaginalis*. The CRS was defined as positive if there was a positive result by either wet mount or culture/PCR. Samples were classified as negative if all comparators were negative. The comparison included the calculation of overall accuracy, sensitivity, specificity, positive predictive value, and negative predictive value according to standard equations. These measures are presented with two-sided 95% Wilson score confidence intervals. Cohen’s kappa coefficient (κ) is presented as a measure of inter-test agreement, with 95% confidence interval. All analyses were performed using Excel (Microsoft Corp. Redmond, Wash.), and SPSS (IBM Corp. IBM SPSS Statistics for Windows. Armonk, NY).

### The investigational test

The GYNI™ system is aimed at providing non-expert health-care providers with the means to obtain a wide diagnostic range, with a fast and inexpensive analysis of various vulvovaginal conditions from a single swab. This is done by fully automating (a) consideration of patient’s reported symptoms (b) saline wet mount microscopy (c) KOH microscopy, and (d) pH measurement, similar to the evaluation performed in vaginitis-specializing clinics^29^. This point of care test does not replace an adequate history taking or physical examination. It does offer a rapid, readily available saline and 10% KOH automated microscopy, which are critical diagnostic steps, without a need for caregiver’s training or any previous skills. It also contains software logic which excludes conditions based on pH or patient’s reported symptoms, according to current related clinical knowledge. The test uses machine learning computer vision in the form of a deep convolutional neural network (CNN) model that performs a multi-label classification of seven major vaginitis conditions. The model was trained on 13,500 microscopy images which were collected and classified by a specialist in a dedicated clinical trial (ClinicalTrials.gov, NCT03585049). Unlike described proofs of concept for applying deep neural networks for the classification of smaller subsets of vaginitis conditions in manually gram-stained and selected microscopy images^30,31^, this test does not use slide staining, therefore, minimizing the required operator labor and shortening the time until results are available.

**Fig. 4:**
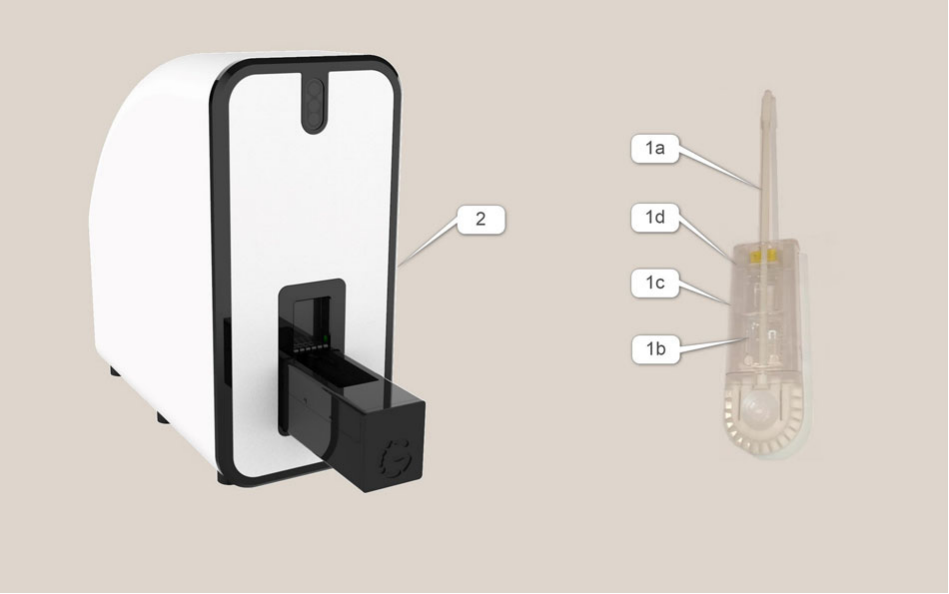
The Gyni investigational test system components. Actual hardware components of the disposable discharge collection tool and the tabletop scanner.

The investigational test system is comprised of the following components (Fig. 4):

#### A vaginal discharge collection tool

A disposable plastic assembly with a swab/brush-head, connected to a plastic rod (1a), that is used to collect the discharge sample from the vagina; (1b) a “floating” transparent optical tray; (1c) transparent cover and; (1d) diluent containers.

#### A compact tabletop scanner

The scanner (2) includes a high-resolution color camera, illumination LEDs, magnifying lenses, and linear motion systems, both vertical and horizontal. The operator places the disposable cartridge in a cradle connected to the horizontal motion system. During insertion of the tray into the cradle, the cartridge’s internal diluents are automatically sprayed on the optic tray and the tray is lifted to create optical coupling. Upon activation, the cradle begins its linear motion between the illumination sources and the camera, dozens of microscopic pictures are acquired, and the pH level is determined by a color change of pH indicator paper located in the cartridge. The collected information is then transferred to the cloud.

#### Web-based user interface

A test operation website provides an interactive mechanism for data entry and test control, available via any web-connected device, such as a laptop or a smartphone.

#### Cloud software platform

Test processing includes a deep learning convolutional network model for multi-label image processing classification, analysis of pH paper images, and color calibration images for pH calculation. The processing software also cross matches the patient’s reported symptoms and pH level with the results of the computer vision classification of the microscopy images.

**Fig. 5:**
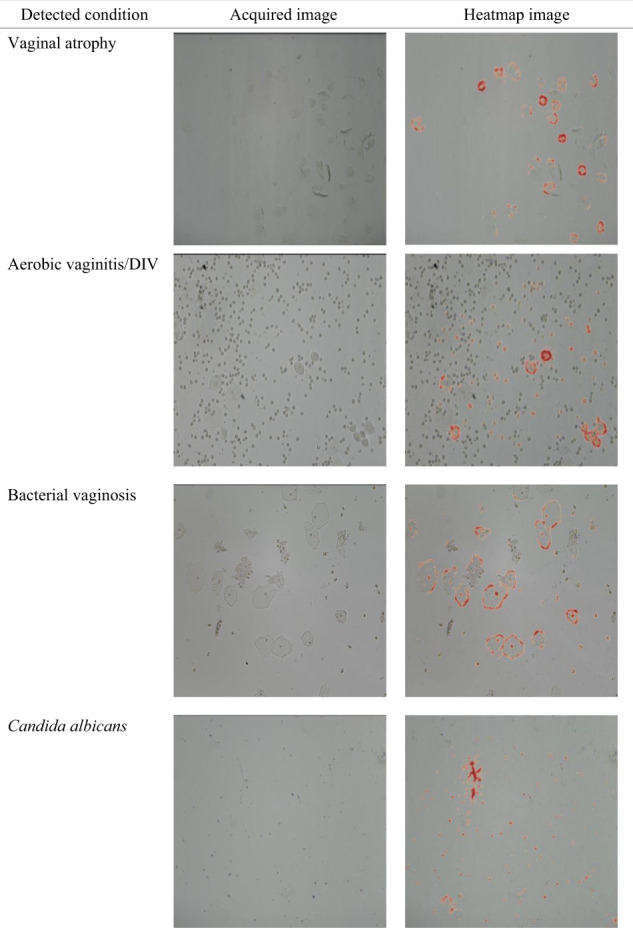
Heatmap examples. Examples for microscopy heatmap images included with the investigational test results.

The results provided by the investigational test include (a) suggested diagnoses–one or more detected conditions, (b) pH level, and (c) a heatmap annotation of a selected input microscopy image, obtained via guided-back propagation^32^. The heatmap annotation is aimed to provide explainability^33^ by visualizing the salient areas in the input image having the strongest effect on the model output. Examples of such heatmap results are shown in Fig. 5. The test results are displayed online and are available to the operator for download.

### Reporting summary

Further information on research design is available in the Nature Research Reporting Summary linked to this article.

### Supplementary information

REPORTING SUMMARY

## Data Availability

The datasets analyzed for the current study are available from the corresponding author on reasonable request. This includes individual de-identified patient data such as patient age, patient-reported symptoms, and lab test results as well as the physician diagnosis and the compared suggested diagnosis by the investigational test.
