# Supplementary material for: Diagnostic performance of an automated microscopy and pH test for diagnosis of vaginitis
Source: NPJ Digit Med. 2023 Apr 13;6:66. doi: 10.1038/s41746-023-00815-w (PMC10102000; doi:10.1038/s41746-023-00815-w)
Supplement: Supplementary file 1 — REPORTING SUMMARY [file 41746_2023_815_MOESM1_ESM.pdf]

nature portfolio

Corresponding author(s): Prof. Ahinoam Lev-Sagie

Last updated by author(s): Jan 31, 2023

Reporting Summary

Nature Portfolio wishes to improve the reproducibility of the work that we publish. This form provides structure for consistency and transparency in reporting. For further information on Nature Portfolio policies, see our Editorial Policies and the Editorial Policy Checklist.

Statistics

For all statistical analyses, confirm that the following items are present in the figure legend, table legend, main text, or Methods section.

n/a	Confirmed	
☐	☒	The exact sample size (*n*) for each experimental group/condition, given as a discrete number and unit of measurement
☐	☒	A statement on whether measurements were taken from distinct samples or whether the same sample was measured repeatedly
☐	☒	The statistical test(s) used AND whether they are one- or two-sided<br>*Only common tests should be described solely by name; describe more complex techniques in the Methods section.*
☒	☐	A description of all covariates tested
☒	☐	A description of any assumptions or corrections, such as tests of normality and adjustment for multiple comparisons
☐	☒	A full description of the statistical parameters including central tendency (e.g. means) or other basic estimates (e.g. regression coefficient) AND variation (e.g. standard deviation) or associated estimates of uncertainty (e.g. confidence intervals)
☒	☐	For null hypothesis testing, the test statistic (e.g. *F*, *t*, *r*) with confidence intervals, effect sizes, degrees of freedom and *P* value noted<br>*Give P values as exact values whenever suitable.*
☒	☐	For Bayesian analysis, information on the choice of priors and Markov chain Monte Carlo settings
☒	☐	For hierarchical and complex designs, identification of the appropriate level for tests and full reporting of outcomes
☐	☒	Estimates of effect sizes (e.g. Cohen's *d*, Pearson's *r*), indicating how they were calculated

Our web collection on statistics for biologists contains articles on many of the points above.

Software and code

Policy information about availability of computer code

Data collection	Microsoft Excel version 2212
Data analysis	IBM SPSS version 29

For manuscripts utilizing custom algorithms or software that are central to the research but not yet described in published literature, software must be made available to editors and reviewers. We strongly encourage code deposition in a community repository (e.g. GitHub). See the Nature Portfolio guidelines for submitting code & software for further information.

Data

Policy information about availability of data

All manuscripts must include a data availability statement. This statement should provide the following information, where applicable:

- Accession codes, unique identifiers, or web links for publicly available datasets
- A description of any restrictions on data availability
- For clinical datasets or third party data, please ensure that the statement adheres to our policy

The datasets analyzed for the current study are available from the corresponding author on reasonable request. This includes individual de-identified patient data such as patient age, patient reported symptoms and lab test results as well as the physician diagnosis and the compared suggested diagnosis by the investigational test.

Human research participants

Policy information about studies involving human research participants and Sex and Gender in Research.

Reporting on sex and gender	The findings apply to only one sex: Female patients
Population characteristics	The findings apply to only one sex: Female patients
Recruitment	The clinic running the study is a referral clinic, with patients attending it for various vulvovaginal complaints, including chronic pain symptoms and dyspareunia. As we wanted to compare the investigational test to specialist's diagnosis regarding vaginitis diagnoses, we invited only those with vaginal symptoms and not those with isolated chronic vulvar complaints, pain and dyspareunia. For this reason, we defined that the study "evaluated women reporting vaginal symptoms" (page 9, line 11), and detailed in inclusion criterion i:" women with vaginal complaints: discharge, malodor, itch, burning, pain, or dryness".
Ethics oversight	Clalit Health Organization Ethics Committee (Approved on Nov 26th, 2019, Approval# 0120-19-COM1, NCT04219605)

Note that full information on the approval of the study protocol must also be provided in the manuscript.

Field-specific reporting

Please select the one below that is the best fit for your research. If you are not sure, read the appropriate sections before making your selection.

☒ Life sciences ☐ Behavioural & social sciences ☐ Ecological, evolutionary & environmental sciences

For a reference copy of the document with all sections, see nature.com/documents/nr-reporting-summary-flat.pdf

Life sciences study design

All studies must disclose on these points even when the disclosure is negative.

Sample size	The sample size for this study was calculated for estimating the overall accuracy (the percent agreement on the diagonal between the investigational test and the reference diagnosis) via the level of precision required for the estimate. The level of precision is measured by the half-width of the 95% confidence interval around the proportion of interest. We calculated based on Hajian-Tilaki K28 that an accuracy level of at least 90% with a confidence interval half-width of 5% can be estimated with a minimum sample size of 139 patients. Recruitment continued to a larger sample size to obtain reasonable representation of each of the included conditions.
Data exclusions	Performance results for vaginitis conditions with insufficient recruitment number were excluded - this applies for T. vaginalis and Candida non albicans vaginitis
Replication	From every recruited patient we collected one swab for in-clinic manual specialist microscopy, one disposable swab of the investigational test, and one was sent for lab tests. Each of those samples was tested once, as there is no available option for retesting them.
Randomization	The order of collection of the three swabs per patient, was random.
Blinding	Yes, the investigators performing microscopy were blinded of the investigational test results, and Lab technicians performing lab tests were blinded of both investigator and investigational test results.

Reporting for specific materials, systems and methods

We require information from authors about some types of materials, experimental systems and methods used in many studies. Here, indicate whether each material, system or method listed is relevant to your study. If you are not sure if a list item applies to your research, read the appropriate section before selecting a response.

Materials & experimental systems

n/a	Involved in the study	
☒	☐	Antibodies
☒	☐	Eukaryotic cell lines
☒	☐	Palaeontology and archaeology
☒	☐	Animals and other organisms
☐	☒	Clinical data
☒	☐	Dual use research of concern

Methods

n/a	Involved in the study	
☒	☐	ChIP-seq
☒	☐	Flow cytometry
☒	☐	MRI-based neuroimaging

Clinical data

Policy information about clinical studies

All manuscripts should comply with the ICMJE guidelines for publication of clinical research and a completed CONSORT checklist must be included with all submissions.

Clinical trial registration	NCT04219605
Study protocol	The full protocol document was included with the submission
Data collection	This prospective cross-sectional study evaluated women reporting vaginal symptoms, seen at a single designated clinic for vulvovaginal disorders at the Ramat-Eshkol women's health clinic of the Clalit Health Organization, Jerusalem, Israel. A total of 226 women with vaginitis symptoms were recruited between December 2020 and October 2022.
Outcomes	The predefined outcome measures were the invastigational test diagnostic performance, i.e., sensitivity and specificity for the different conditions: BV/Candida albicans/Candida non albicans/Atrophy/Cytolytic vaginosis/DIV/Trichomonas/Normal discharge) compared to defined gold Standard of Care (SOC) .<br>Outcome evaluation: The results of the investigational test were compared individually to the CRS comparator of the specialist wet mount results, the candida cultures, and the STI PCR panel for detection of T. vaginalis. The CRS was defined as positive if there was a positive result by either wet mount or culture/PCR. Samples were classified as negative if all comparators were negative. The comparison included the calculation of overall accuracy, sensitivity, specificity, positive predictive value, and negative predictive value according to standard equations. These measures are presented with two-sided 95% Wilson score confidence intervals.
